# Ca^2+^-dependent endoplasmic reticulum stress correlation with astrogliosis involves upregulation of KCa3.1 and inhibition of AKT/mTOR signaling

**DOI:** 10.1186/s12974-018-1351-x

**Published:** 2018-11-15

**Authors:** Zhihua Yu, Fangfang Dou, Yanxia Wang, Lina Hou, Hongzhuan Chen

**Affiliations:** 1 Department of Pharmacology and Chemical Biology, Shanghai Jiao Tong University School of Medicine, 280 South Chongqing Road, Shanghai, 200025 China; 2 Shanghai University of Traditional Chinese Medicine, Shanghai, 201203 China; 3 Basic Research Department, Shanghai Geriatric Institute of Chinese Medicine, Shanghai, 200031 China; 4 Experimental Teaching Center of Basic Medicine, Shanghai Jiao Tong University School of Medicine, Shanghai, 200025 China

**Keywords:** Alzheimer’s disease, Endoplasmic reticulum stress, Mouse model, Unfolded protein response, KCa3.1

## Abstract

**Background:**

The intermediate-conductance Ca^2+^-activated K^+^ channel KCa3.1 was recently shown to control the phenotype switch of reactive astrogliosis (RA) in Alzheimer’s disease (AD).

**Methods:**

KCa3.1 channels expression and cell localization in the brains of AD patients and APP/PS1 mice model were measured by immunoblotting and immunostaining. APP/PS1 mice and KCa3.1^−/−^/APP/PS1 mice were subjected to Morris water maze test to evaluate the spatial memory deficits. Glia activation and neuron loss was measured by immunostaining. Fluo-4AM was used to measure cytosolic Ca^2+^ level in β-amyloid (Aβ) induced reactive astrocytes in vitro.

**Results:**

KCa3.1 expression was markedly associated with endoplasmic reticulum (ER) stress and unfolded protein response (UPR) in both Aβ-stimulated primary astrocytes and brain lysates of AD patients and APP/PS1 AD mice. The KCa3.1 channel was shown to regulate store-operated Ca^2+^ entry (SOCE) through an interaction with the Ca^2+^ channel Orai1 in primary astrocytes. Gene deletion or pharmacological blockade of KCa3.1 protected against SOCE-induced Ca^2+^ overload and ER stress via the protein kinase B (AKT) signaling pathway in astrocytes. Importantly, gene deletion or blockade of KCa3.1 restored AKT/mechanistic target of rapamycin signaling both in vivo and in vitro. Consistent with these in vitro data, expression levels of the ER stress markers 78-kDa glucose-regulated protein and CCAAT/enhancer-binding protein homologous protein, as well as that of the RA marker glial fibrillary acidic protein were increased in APP/PS1 AD mouse model. Elimination of KCa3.1 in KCa3.1^−/−^/APP/PS1 mice corrected these abnormal responses. Moreover, glial activation and neuroinflammation were attenuated in the hippocampi of KCa3.1^−/−^/APP/PS1 mice, as compared with APP/PS1 mice. In addition, memory deficits and neuronal loss in APP/PS1 mice were reversed in KCa3.1^−/−^/APP/PS1 mice.

**Conclusions:**

Overall, these results suggest that KCa3.1 is involved in the regulation of Ca^2+^ homeostasis in astrocytes and attenuation of the UPR and ER stress, thus contributing to memory deficits and neuronal loss.

## Introduction

Alzheimer’s disease (AD) is a neurodegenerative disorder leading to a progressive decline in cognitive function that is characterized at the molecular level by β-amyloid (Aβ)-induced synaptic dysfunction, neuronal loss, tau pathology, and oxidative stress (Verkhratsky et al. 2010). Reactive gliosis, including microglia activation and astrocyte reactivation, plays a significant role in the pathogenesis of AD, both in transgenic rodent models and human patients [1, 2]. During the onset of AD, abnormal Aβ metabolism has a profound effect on the local community of neurons and glial cells in the central nervous system (CNS), while in the developmental stage of disease, the inflammatory response of reactive glial cells contributes to neuronal Ca^2+^ dysregulation.

In neurons, Aβ peptides induce neurotoxic effects that are mediated via deregulation of intracellular calcium ([Ca^2+^]_i_) homeostasis, which results in synaptic loss. However, little is known about the role of Aβ in the regulation of astrocytic Ca^2+^ homeostasis and subsequent pathological influences. Stimulation of Aβ in primary astrocytes triggers elevations in [Ca^2+^]_i_ and the formation of reactive oxygen species, which are associated with metabolic failure of astrocytes and may directly induce neuronal loss [3–5]. Notably, spontaneous [Ca^2+^]_i_ accumulation has been identified in astrocytes of amyloid precursor protein/presenilin 1 (APP/PS1) mice in vivo, independent of neuron hyperactivity [6].

The endoplasmic reticulum (ER) regulates [Ca^2+^]_i_ homeostasis and protein folding. Store-operated Ca^2+^ entry (SOCE) channels, which are complexes composed of stromal interaction molecule 1 (STIM1, an ER calcium sensor) and calcium release-activated calcium channel protein 1 (Orai1, a pore-forming protein), can be activated by the depletion of Ca^2+^ stores in the ER [7]. SOCE plays an essential role in the activation of non-excitable cells, including astrocytes and microglia, via triggering Ca^2+^ influx [8]. Disruption of protein folding in the ER triggers the unfolded protein response (UPR) via activation of three ER pathways: inositol-requiring enzyme 1 (IRE1), activating transcription factor 6 (ATF6), and PKR-like ER kinase (PERK) [9, 10]. The 78-kDa glucose-regulated protein (GRP78) dissociates from PERK, ATF6, and IRE1 during ER stress, and then initiates proapoptotic signaling via activation of the CCAAT/enhancer-binding protein homologous protein (CHOP). Accumulating evidence suggests that Ca^2+^-dependent ER stress is correlated with reactive astrogliosis (RA) in both an AD mouse model and ischemic stroke [11, 12]. In addition, the intermediate-conductance Ca^2+^-activated K^+^ channel KCa3.1 is reported to be involved in cisplatin-initiated acute kidney injury [13] and the phenotypic switch of RA in ischemic stroke [12] via attenuation of ER stress.

KCa3.1 regulates K^+^ efflux and subsequent Ca^2+^ entry via hyperpolarization of the membrane potential [14]. The KCa3.1 channel has been investigated as a therapeutic target in neurodegeneration diseases, vascular restenosis, and autoimmune diseases [15–17]. Our group recently reported that KCa3.1 inhibition significantly ameliorated neuronal loss, RA, microglial activation, and memory deficits in both APP/PS1 mice and senescence-accelerated mouse prone 8 (SAMP8) mice [15, 18]. KCa3.1 also plays a key role in mediating Aβ-induced RA, which includes activation of the mitogen-activated protein kinase/c-Jun N-terminal kinase (MAPK/JNK) signaling pathway and upregulation of reactive oxygen species (ROS) production. Blockade of KCa3.1 channels with triarylmethane-34 (TRAM-34) was shown to attenuate microglial-dependent indirect neurotoxicity in vivo, which emphasizes the role of Ca^2+^ in the process of neuroinflammation [19, 20]. Collectively, these data clearly demonstrate that KCa3.1 presents a potential therapeutic target in AD.

Therefore, the aim of present study was to elucidate the underlying mechanisms of Aβ-mediated ER stress in RA and to identify key molecules involved in the regulation of RA-induced neurotoxicity. We report that Ca^2+^ overload induces ER stress in primary astrocytes and ER stress is increased in AD patients and APP/PS1 AD mice. Blockade or gene deletion of KCa3.1 decreased SOCE-induced Ca^2+^ overload and attenuated ER stress via the protein kinase B/mechanistic target of rapamycin (AKT/mTOR) pathway in primary astrocytes, which is essential to protect neurons against RA-induced neurotoxicity. We also show that the absence of KCa3.1 channels attenuated ER stress, gliosis, neuroinflammation, memory deficits, and neuronal loss in KCa3.1^−/−^/APP/PS1 mice. These results reveal an important correlation between KCa3.1 activation and cognitive deficits in APP/PS1 AD mice, suggesting that KCa3.1 may be an effective therapeutic target in AD.

## Materials and methods

### Brain autopsy material

Frozen tissues and paraffin-embedded brain slices from the hippocampi of postmortem human samples of control and AD patients were obtained from the Netherlands Brain Bank (Netherlands Institute for Neuroscience, Amsterdam, Netherlands). The Netherlands Brain Bank obtained written informed consent for brain autopsy specimens for research purposes after death. The Braak stage, neuropathological and clinical diagnoses, age and gender distributions, as well as diagnostic groupings are presented in Table 1. The frozen tissues were used to isolate proteins, and expression of proteins was evaluated by Western blotting as described below. Immunofluorescence was performed on 8-μm sections using antibodies against glial fibrillary acidic protein (GFAP), KCa3.1, Orai1, and GRP78 antibodies as described below.

**Table 1:** Demographic data of the cases studied

Diagnosis	Gender	Age	Braak stage	Amyloid	APOE	Analysis
Control-1	F	73	0	B	E3/E2	WB, IF
Control-2	M	99	2	C	–	WB, IF
Control-3	F	81	1	O	E3/E3	WB, IF
Control-4	F	89	2	B	–	WB, IF
Control-5	M	84	2	A	–	WB, IF
Control-6	F	64	0	A	E3/E2	WB, IF
AD-1	F	81	6	C	E4/E3	WB, IF
AD-2	F	62	6	C	E4/E3	WB, IF
AD-3	M	84	6	C	–	WB, IF
AD-4	F	72	6	C	E3/E2	WB, IF
AD-5	F	89	6	C	E4/E3	WB, IF
AD-6	M	96	6	C	–	WB, IF

### Animals

Wild-type (WT) C57BL/6 mice (male, 25–30 g) were procured from the Shanghai Laboratory Animal Center (Shanghai, China). KCa3.1^−/−^ mice were obtained from the Jackson Laboratory (Bar Harbor, ME, USA) and housed as described previously [18]. APP/PS1 transgenic mice (an AD model) were also purchased from the Jackson Laboratory (no. 003378) [15]. KCa3.1^−/−^ mice were crossed with APP/PS1 mice and the offspring were intercrossed to generate mice with the KCa3.1^−/−^/APP/PS1 genotype. Mouse cohorts of the four genotypes (WT, KCa3.1^−/−^, KCa3.1^−/−^/APP/PS1, and APP/PS1) were generated and used for behavioral analysis. The protocols of all animal experiments were approved by the Animal Experimentation Ethics Committee of Shanghai Jiao Tong University School of Medicine, Shanghai, China (ethics protocol number: A-2015-010).

### Morris water maze test

The modified Morris water maze (MWM) test was performed as previously described [21]. Briefly, the test requires the mice to receive 5 consecutive training days (1 day with the visible platform and 4 days with the hidden platform) in a circular water tank (diameter = 80 cm; height = 50 cm) containing opaque water (22 °C, 25 cm deep). A spatial probe trial was performed on day 6 with no platform present. During the hidden platform training days, the mice were allowed to swim for 60 s to find the hidden platform (1 cm below the water surface). The platform was removed from the pool on day 6 of the spatial probe trial and the mice swam freely for 60 s. Performance of the MWM test was recorded using a video camera and analyzed using a video tracking system (Jiliang Software Technology Co., Ltd., Shanghai, China).

### Immunostaining and data analysis

Confocal microscopy was performed as previously described [18]. Briefly, mice were anesthetized with chloral hydrate and perfused with 4% paraformaldehyde. Then, 10% goat serum in 0.01 M phosphate-buffered saline (PBS) was used to block 12-μm sections of the mice brain or 8-μm sections of the human brain for 1 h at room temperature. The brain sections were then incubated with the following primary antibodies: rabbit anti-GFAP (1:1000; Dako, Glostrup, Denmark), mouse anti-GFAP (1:200; Merck Millipore, Burlington, MA, USA), rabbit anti-NeuN (1:100; Merck Millipore, Burlington, MA, USA), rabbit anti-Iba1 (1:500; Wako Pure Chemical Industries, Ltd., Osaka, Japan), mouse anti-KCa3.1 (1:100; Alomone Labs, Ltd., Jerusalem, Israel), rabbit anti-GRP78 (1:100; Cell Signaling Technology, Inc., Beverly, MA, USA), mouse anti-NG2 (Sigma-Aldrich Corporation, St. Louis, MO, USA), and rabbit anti-Orai1 (1:100; Santa Cruz Biotechnology, Inc., Dallas, TX, USA). After incubation at 4 °C overnight, the brain sections were incubated with the respective Alexa Fluor® 488- or 568-conjugated secondary antibodies (1500; Invitrogen Corporation, Carlsbad, CA, USA). Images were collected using a TCS SP8 confocal laser scanning microscope (Leica Microsystems, Wetzlar, Germany) and processed with Leica LASAF Lite imaging software. The same reference position was used for each brain slice for quantification, i.e., between three and five random 0.01-mm^2^ microscopic fields. Quantification was measured from six sections per brain (120-μm intervals) in a blinded manner.

### Western blot analysis

Mice were anesthetized with chloral hydrate and perfused with saline. Mice or human brain tissues and cells were lysed on ice with radioimmunoprecipitation assay buffer (50 mM Tris (pH 7.4), 150 mM NaCl, 1% Triton X-100; 1% sodium deoxycholate, 0.1% sodium dodecyl sulfate, sodium orthovanadate, sodium fluoride, ethylenediaminetetraacetic acid, leupeptin) containing 1% phenylmethanesulfonyl fluoride. The supernatants were collected after centrifuging at 13,500 rpm for 5 min at 4 °C. Equal concentrations of proteins were separated by 10% (*w*/*v*) sodium dodecyl sulfate-polyacrylamide gel electrophoresis (SDS-PAGE) and then transferred to a polyvinylidene difluoride membrane, which was blocked for 1 h at room temperature (RT) in 5% milk in Tris-buffered saline with 0.05% Tween 20. The membrane was incubated with the following primary antibodies overnight at 4 °C: mouse anti-KCa3.1 (1:100; Alomone Labs, Ltd.), mouse anti-STIM1 (1:500; Santa Cruz Biotechnology, Inc.), rabbit anti-Orai1 (1:1000; Santa Cruz Biotechnology, Inc.), rabbit anti-GRP78, rabbit anti-mTOR, rabbit anti-phospho-mTOR (Ser2448), rabbit anti-phospho-PERK (Thr980), rabbit anti-phospho-eIF2α, rabbit anti-phospho-Akt (Thr308), rabbit anti-Akt, rabbit anti-phospho-4E-BP1, rabbit anti-phospho-p70 S6 Kinase, mouse anti-CHOP, rabbit anti-iNOS, rabbit anti-COX2 (1:1000; Cell Signaling Technology), and mouse anti-β-actin (1:1000; Beyotime Institute of Biotechnology, Haimen, China). The membranes were subsequently washed and incubated with anti-rabbit or anti-mouse HRP for 1 h at RT, and developed using BeyoECL solution (P0018A; Beyotime Institute of Biotechnology). Image Studio Lite Ver 5.2 software (LI-COR Biosciences, Lincoln, NE, USA) was used to analyze the proteins.

### Enzyme-linked immunosorbent assay

ELISA was performed using the kit for TNF-α and IL-1β (Rapidbio Labs, Langka Trade Co. Ltd., Shanghai, China). The procedures were conducted according to manufacturer’s protocols.

### Preparation of oligomeric Aβ peptides

The oligomeric Aβ was prepared as described previously (Yi et al. 2016b). Briefly, monomeric peptide Aβ was dissolved in 1,1,1,3,3,3-hexafluoro 2-propanol (Sigma-Aldrich Corporation, St. Louis, MO, USA) at 1 mg/mL. The dried monomeric peptide Aβ was resolved in ddH_2_O at a concentration of 100 mM and incubated at 4 °C for 24 h to induce aggregation.

### Primary cultures

Primary cortical astrocyte cultures derived from newborn (P0–P2) WT or KCa3.1^−/−^ C57BL/6 mouse brains were prepared from mixed glial cultures (10–14 days in vitro) as described previously [22]. Briefly, the cerebral cortices were dissociated into single cell suspensions. Dissociated single cells were grown in Dulbecco’s modified Eagle’s medium supplemented with 10% fetal bovine serum at 37 °C under an atmosphere of 5% CO_2_. When the astrocytes grew to confluence (10–14 days later), the flasks were shaken overnight (200 rpm, 37 °C) to deplete microglia and oligodendrocytes. The purified astrocytes were then plated onto 6-well or 96-well plates in serum containing medium. Astrocytes were incubated with serum-free DMEM for 24 h after reaching confluence again**,** and then were treated with Aβ for different time periods before harvest. The culture medium (CM) was changed to neurobasal medium (NB) with B27 supplement (Invitrogen Corporation) after confluent astrocytes were serum-free for 24 h. The NB/B27-based WT and KCa3.1^−/−^ astrocytes were then treated with 5 μM Aβ oligomer. The CM from the NB/B27-based cells was collected and used immediately. The primary cultured cortical neurons were isolated from neonatal (P0–P2) C57BL/6 mice and cultured as described previously (Yi et al. 2016b).

### Neurite outgrowth assay

Cells were stained with primary antibody against microtubule-associated protein 2 (MAP2) and Alexa Fluor® 568-conjugated secondary antibody. A Cellomics Kinetic Scan reader (Thermo Fisher Scientific, Waltham, MA, USA) was used to scan the MAP2-positive cells. Extended Neurite Outgrowth software (Thermo Fisher Scientific) was used to analyze the data.

### Calcium imaging

The purified astrocytes were plated in the wells of 96-well plates containing DMEM supplemented with 10% fetal bovine serum. After once again reaching confluence, the cells were incubated with the fluorescent calcium indicator Fluo-4AM (1.6 μM; Beyotime Institute of Biotechnology) for 25 min and then washed three times with 0.01 M PBS. Afterward, 2 μM ethylene glycol-bis (β-aminoethyl ether)-*N*,*N*,*N*′,*N*′-tetraacetic acid was used to chelate the calcium in the DMEM and then 1 μM thapsigargin (Tg) was used to induce [Ca^2+^]_i_ release. After the [Ca^2+^]_i_ concentration was stable, 2 mM CaCl_2_ solution was added to the DMEM to induce Ca^2+^ influx. Fluorescence signals were recorded and analyzed using a FlexStation 3 Multi-Mode Microplate Reader (Molecular Devices, Sunnyvale, CA, USA).

### Statistical analysis

All data are presented as the mean ± standard error of the mean. Statistical analyses were performed using Prism software (GraphPad Software, Inc., La Jolla, CA, USA). Data were tested for Gaussian distribution with the Kolmogorov–Smirnov normality test and then analyzed by one-way analysis of variance (ANOVA) and the Dunnett’s post-hoc test. Data were analyzed with an unpaired, two-tailed Student’s *t* test when comparing between two groups or the non-parametric Mann–Whitney test was applied. Statistical significance was set at *p* < 0.05.

## Results

### Ca^2+^ overload induced ER stress in primary astrocytes and ER stress is increased in AD patients and APP/PS1 AD mice

**Fig. 1:**
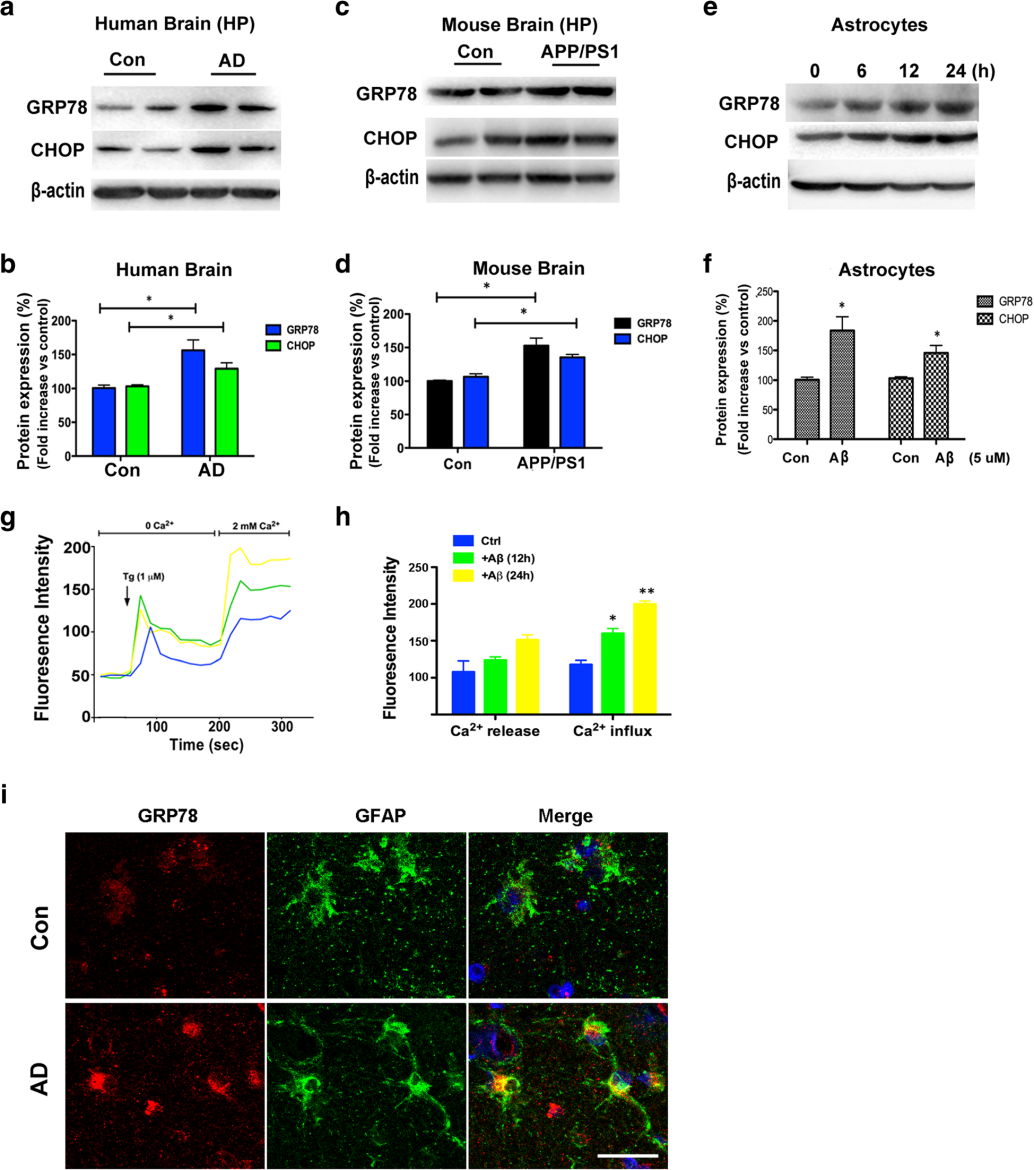
Aβ induced intracellular Ca^2+^ overload by increasing SOCE, which resulted in ER stress. **a** Representative blots of GRP78 and CHOP from the hippocampi of postmortem human AD and age-matched controls. **b** Data are presented as the mean ± SEM (*n* = 5). The optical density (OD) values of GRP78 and CHOP were normalized to that of β-actin. *p < 0.05 vs. control brains (unpaired, two-tailed Student’s *t* test). **c** Representative blots of GRP78 and CHOP from the hippocampi of APP/PS1 mice and age-matched controls. **d** Data are presented as the mean ± SEM (*n* = 5). The OD values of GRP78 and CHOP were normalized to that of β-actin. *p < 0.05 vs. control brains (unpaired, two-tailed Student’s *t* test). **e** Primary astrocytes were treated with 5 μM Aβ for 6, 12, or 24 h. Then, the cell lysates were subjected to western blot analysis with β-actin as the loading control. **f** Data are presented as the mean ± SEM (*n* = 3). The OD values of GRP78 and CHOP were normalized to that of β-actin. *p < 0.05 vs. control cells (unpaired, two-tailed Student’s *t* test). **g** Primary cultured astrocytes were treated with 5 μM Aβ for 12 or 24 h and then loaded with the Ca^2+^ sensitive dye Fluo-4AM at 37 °C for 30 min. Changes in [Ca^2+^]_i_ were monitored with a FlexStation 3 Multi-Mode Microplate Reader. Fluorescence intensities of [Ca^2+^]_i_ are shown. Fluorescence intensity was measured in the presence of 1 μM Tg with or without 2 mM Ca^2+^. *Tg* thapsigargin. **h** Quantification (mean ± SEM) of fluorescence intensity. *p < 0.05, **p < 0.01 vs. control cells (one-way ANOVA followed by the Dunnett’s multiple comparison test). **i** Upregulation of GRP78 in reactive astrocytes of AD patients. Double immunofluorescence staining of GRP78 with GFAP in brain sections of control and AD patients. Nuclei were stained blue with 4′,6-diamidino-2-phenylindole (DAPI). Scale bar: 25 μm

ER stress induced by increased protein misfolding and upregulation of the UPR has been observed in AD patients and in an AD mouse model [23, 24]. Disruption of protein folding in the ER triggers the UPR via activation of three ER pathways: PERK, ATF6, and IRE1 [9, 10]. The GRP78 dissociates from PERK, ATF6, and IRE1 during ER stress, and then initiates proapoptotic signaling via activation of CHOP. GRP78 and CHOP protein levels were increased in both the brains of AD patients (*p* < 0.05, Fig. 1a, b) and the hippocampi of APP/PS1 mice (*p* < 0.05, Fig. 1c, d), as compared to control tissues. The Aβ oligomer was reported to disrupt Ca^2+^ homeostasis in the ER of astrocytes, which leads to ER stress-induced RA [25]. The addition of Aβ to primary cultured astrocytes significantly upregulated the protein levels of GRP78 and CHOP at 12 h, as compared with control cells, indicating that Aβ induces the activation of the UPR in astrocytes (*p* < 0.05, Fig. 1e, f). Overall, these data obtained from AD patients and APP/PS1 AD mouse models demonstrate a potential mechanism of ER stress activation in AD.

For evaluation of SOCE, Tg (1 μM), an ER Ca^2+^ ATPase pump blocker, was used to deplete ER Ca^2+^ stores. Without extracellular Ca^2+^ (0 Ca^2+^), Tg-induced upregulation of [Ca^2+^]_i_ (first peak) showed no significant changes in response to treatment with 5 μM Aβ at 12 h, as compared to the control astrocytes (*p* > 0.05, Fig. 1g, h). However, SOCE, initiated by external Ca^2+^ (2 mM), was increased at 12 h (*p* < 0.05, Fig. 1g, h) and 24 h (*p* < 0.01, Fig. 1g, h) in response to treatment with 5 μM Aβ, as compared to control cells. Colocalization experiments were conducted using antibodies against the ER stress marker GRP78 and the RA marker GFAP. Double-labeled staining showed that GRP78 and GFAP colocalized in the AD brain tissues, but not the controls (Fig. 1i).

### Aβ increases SOCE by upregulating KCa3.1 expression

**Fig. 2:**
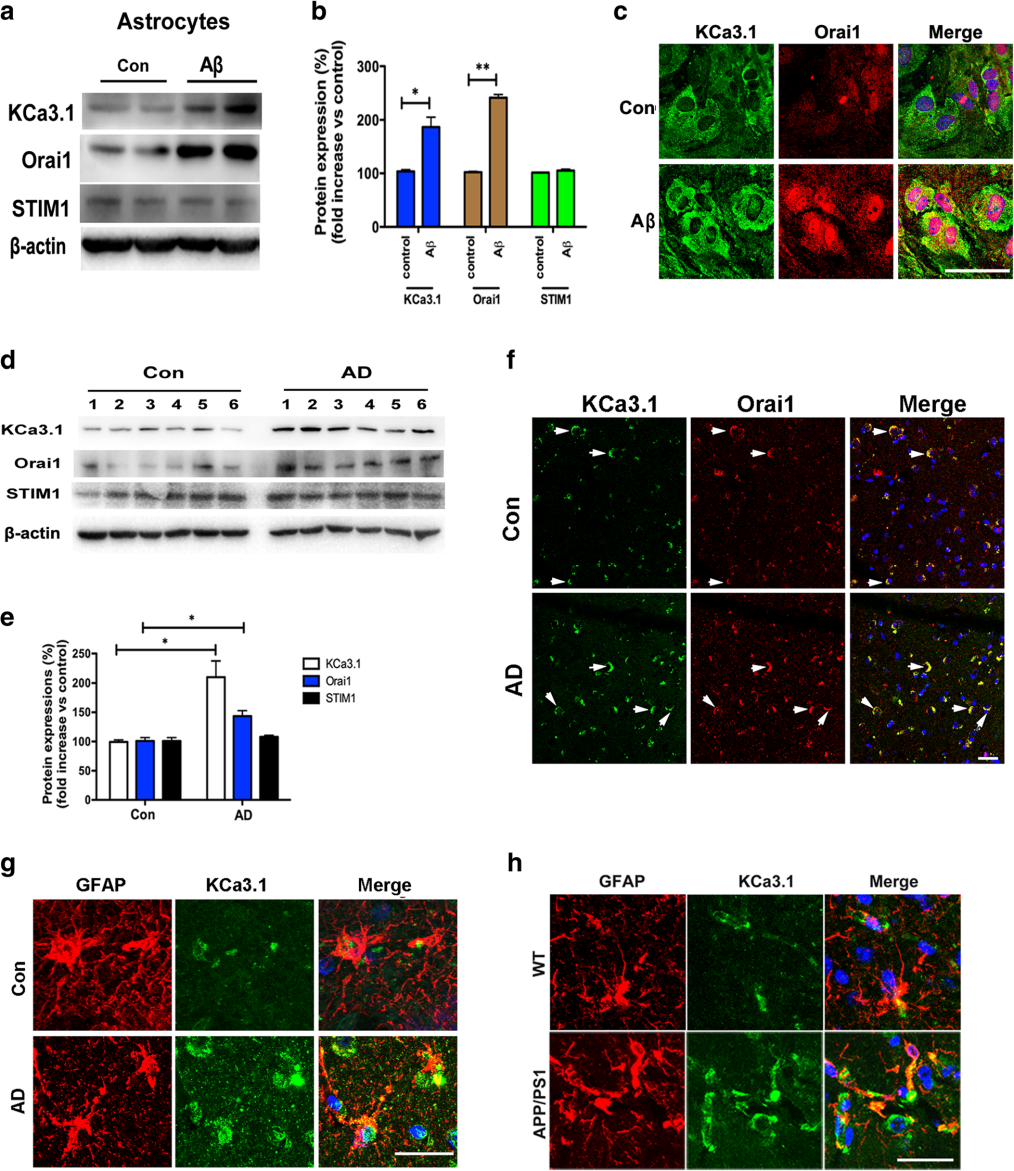
KCa3.1 upregulation in Aβ-induced RA and the brains of AD patients. **a** Primary astrocytes were stimulated with 5 μM Aβ and lysates were subjected to Western blot analysis with antibodies against KCa3.1, Orai1, and STIM1. β-actin was used to confirm equal loading. **b** Data are presented as the mean ± SEM (*n* = 5). The OD values of KCa3.1, Orai1, and STIM1 were normalized to that of β-actin. *p < 0.05 vs. control cells (unpaired, two-tailed Student’s *t* test). **c** Double immunofluorescence staining of KCa3.1 with Orai1 in astrocytes with or without stimulation of 5 μM Aβ. Nuclei were stained in blue with DAPI. Scale bar: 25 μm. **d** Brain lysates from control and AD samples were subjected to SDS-PAGE and immunoblotted with antibodies against KCa3.1, Orai1, and STIM1. **e** Data are presented as the mean ± SEM (*n* = 5). The OD values of KCa3.1, Orai1, and STIM1 were normalized to that of β-actin. *p < 0.05 vs. controls (one-way ANOVA followed by the Dunnett’s multiple comparison test). **f** Double immunofluorescence staining of KCa3.1 with Orai1 in the brain sections of control and AD patients. Arrows indicate colabeling of KCa3.1 with Orai1. Nuclei were stained blue with DAPI. **g**, **h** Double immunofluorescence staining of KCa3.1 with GFAP in the brain sections of control and AD patients (**g**) or WT and APP/PS1 mice (**h**). Scale bar: 50 μm

The accumulation of Aβ oligomers and amyloid plaques in the brain is associated with neuroinflammation and abnormal gliosis, including reactive astrocytes [3, 26]. We previously reported that KCa3.1 gene deletion attenuated ER stress in RA via the c-Jun/JNK and MAPK/ERK signaling pathways in ischemic mice [12]. Given the key role of Aβ-induced astrocytes ER stress due to SOCE-induced Ca^2+^ overload, we then measured the expression levels of KCa3.1 and SOCE channels following stimulation by prolonged Aβ treatment. Although the exact SOCE molecular components in astrocytes are unknown, KCa3.1 and Orai1 have been reported as components of SOCE channels in some cell types [8, 27, 28]. Western blot analysis was performed to evaluate changes in the expression profiles of KCa3.1, Orai1, and STIM1, which revealed upregulation of the KCa3.1 and Orai1 channels in response to stimulation with 5 μM Aβ for 24 h, as compared to the control group, whereas there was no obvious change in the expression of STIM1 (a regulator of KCa3.1 and Orai1) (*p* > 0.05, Fig. 2a, b). Colocalization experiments were conducted using specific KCa3.1 and Orai1 antibodies. Double-labeled staining showed that the colocalization of KCa3.1 and Orai1 was increased in primary astrocytes in response to stimulation with 5 μM Aβ for 24 h, as compared to the control cells (Fig. 2c).

We then further studied the potential relevance of KCa3.1 and Orai1 in the hippocampi of control and AD patients. The results showed that the expression levels of the KCa3.1 and Orai1 proteins, but not STIM1, were upregulated in the hippocampi of AD patients, as compared to the control tissues (*p* < 0.05, Fig. 2d, e). Colocalization experiments were conducted by immunostaining the brain tissues of AD patients and controls with specific KCa3.1 and Orai1 antibodies. Double-labeled staining showed that the colocalization of KCa3.1 and Orai1 was increased in the brain tissues of AD patients, as compared to the controls (Fig. 2f). In the present study, colocalization between KCa3.1 and astrocytes was detected in the brains of AD patients and 15-month-old APP/PS1 mice. Age-matched control humans and WT littermates were used as controls. In AD patients (Fig. 2g) and APP/PS1 mice (Fig. 2h), colocalization of KCa3.1 and GFAP^+^ astrocytes was increased, as compared to the controls.

### Blockade or gene deletion of KCa3.1 decreased SOCE-induced Ca^2+^ overload and attenuated ER stress in primary astrocytes

**Fig. 3:**
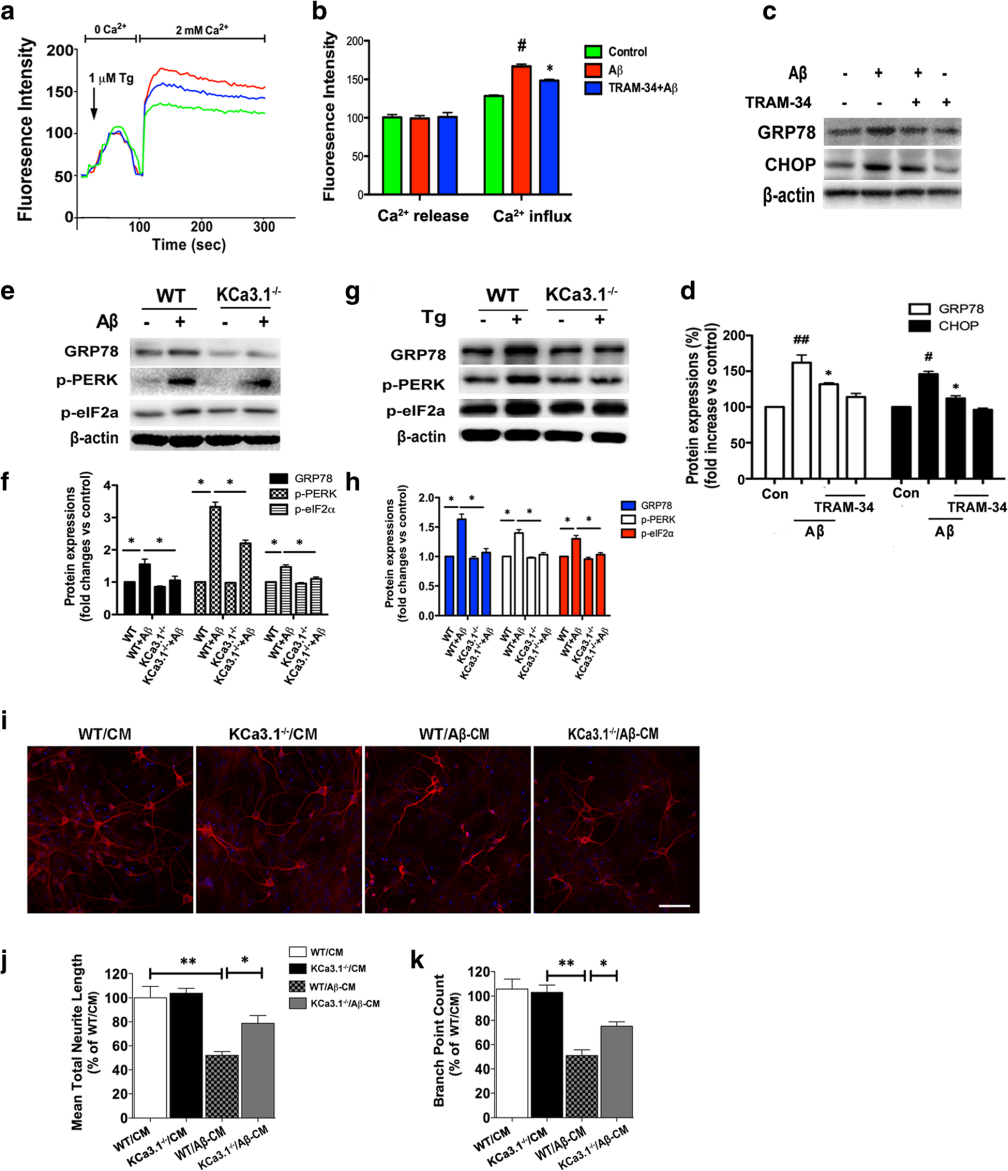
KCa3.1 involved in astrocytes SOCE and ER stress. **a** Primary cultured astrocytes were treated with 5 μM Aβ for 12 h with or without pretreatment of the KCa3.1 blocker TRAM-34 (1 μM). Fluorescence intensities of [Ca^2+^]_i_ are shown. Fluorescence intensity was measured in the presence of 1 μM Tg with or without 2 mM Ca^2+^. **b** Data are presented as the mean ± SEM (*n* = 10). #*p* < 0.05 vs. control, *p < 0.05 vs. Aβ-treated cells. **c** Astrocytes were treated with 5 μM Aβ for 24 h with or without 1 μM TRAM-34 pretreatment, and then subjected to western blot analysis with antibodies against GRP78 and CHOP. **d** Data are presented as the mean ± SEM (*n* = 3). #*p* < 0.05, ##*p* < 0.01 vs. controls, *p < 0.05 vs. Aβ-treated cells. One-way ANOVA followed by the Dunnett’s multiple comparison test vs. control cells. **e**, **g** Representative images of GRP78, p-PERK, and phosphorylated eIF2α (p-eIF2α) in KCa3.1^−/−^ astrocytes, responses to 5 μM Aβ (**e**) or 1 μM Tg (**g**) vs. WT cells. **f**, **h** Mean values of GRP78, p-PERK, and p-eIF2α relative to β-actin. Data are presented as the mean ± SEM (*n* = 3). *p < 0.05 (unpaired, two-tailed Student’s *t* test). **i**–**k** Levels of the dendritic marker MAP2 were compared between neurons treated with CM from WT astrocytes (WT/CM), CM from KCa3.1^−/−^ astrocytes (KCa3.1^−/−^/CM), CM from 5 μM Aβ stimulated WT astrocytes (WT/Aβ-CM), or CM from 5 μM Aβ stimulated KCa3.1^−/−^ astrocytes (KCa3.1^−/−^/Aβ-CM). **i** Neuron dendrites were immunostained with MAP2 and nuclei were stained with DAPI (blue). Scale bars: 25 μm. Neurite length (**j**) and branch point counts (**k**) were analyzed by extended neurite outgrowth bioapplication software. Data represent mean ± SEM (*n* = 3). *p < 0.05, **p < 0.001 (one-way ANOVA followed by the Dunnett’s multiple comparison test). *Tg* thapsigargin, *Con* control, *WT* wild-type

The findings of the above experiments suggest that KCa3.1 plays a key role during the process of SOCE and in maintaining Ca^2+^ homeostasis in the ER. Pharmacological blockade of KCa3.1 was then conducted to further confirm the critical role of KCa3.1 in Ca^2+^ homeostasis and stress in the ER. The results showed that blockade of KCa3.1 with 1 μM TRAM-34 attenuated Aβ-induced SOCE-induced Ca^2+^ overload, as compared with Aβ-treated cells (*p* < 0.05, Fig. 3a, b). Pharmacological blockade of KCa3.1 with 1 μM TRAM-34 decreased the upregulation of GRP78 and CHOP, which was induced by treatment with 5 μM Aβ for 24 h, indicating that blockade of KCa3.1 could inhibit prolonged UPR activation (*p* < 0.05, Fig. 3c, d). KCa3.1 gene deletion (KCa3.1^−/−^) from astrocytes was stimulated with 5 μM Aβ or 1 μM Tg for 24 h, as compared to WT control cells. KCa3.1 gene deletion also decreased Aβ or Tg-induced upregulation of GRP78 (*p* < 0.05, Fig. 3e–h). As a critical transducer of the UPR, phosphorylation of PERK (p-PERK) was increased after stimulation with 5 μM Aβ or 1 μM Tg, but decreased in KCa3.1^−/−^ astrocytes (*p* < 0.05, Fig. 3e–h). Similarly, prolonged UPR has been shown to activate eukaryotic initiation factor 2α (eIF2α), an important downstream signaling target, which was also increased in Aβ or Tg-treated WT astrocytes, but was restored to normal in KCa3.1^−/−^ cells (*p* < 0.05, Fig. 3e–h).

WT and KCa3.1^−/−^ astrocytes were used to test whether KCa3.1-mediated astrogliosis could induce dendritic damage. More dendritic damage was observed in the CM from 5 μM Aβ-stimulated WT astrocytes (WT/Aβ-CM) than in those treated with CM from WT astrocytes (WT/CM), as shown by immunofluorescent staining of the dendritic marker MAP2 (Fig. 3i). Incubation with WT/Aβ-CM decreased total neurite length (*p* < 0.01, Fig. 3j) and number of branch points (*p* < 0.01, Fig. 3k). Incubation with CM from 5 μM Aβ-stimulated KCa3.1^−/−^ astrocytes (KCa3.1^−/−^/Aβ-CM) attenuated the effect of WT/Aβ-CM by increasing total neurite length and the number of branch points (*p* < 0.05, Fig. 3j, k).

### Levels of ER stress were decreased in the brains of APP/PS1 mice lacking KCa3.1

In the present study, APP/PS1 mice were crossed with KCa3.1^−/−^ mice to study the role of KCa3.1 channels in RA-mediated neuronal toxicity. Offspring intercrossing generated mice with the WT, KCa3.1^−/−^, APP/PS1, and KCa3.1^−/−^/APP/PS1 genotypes.

**Fig. 4:**
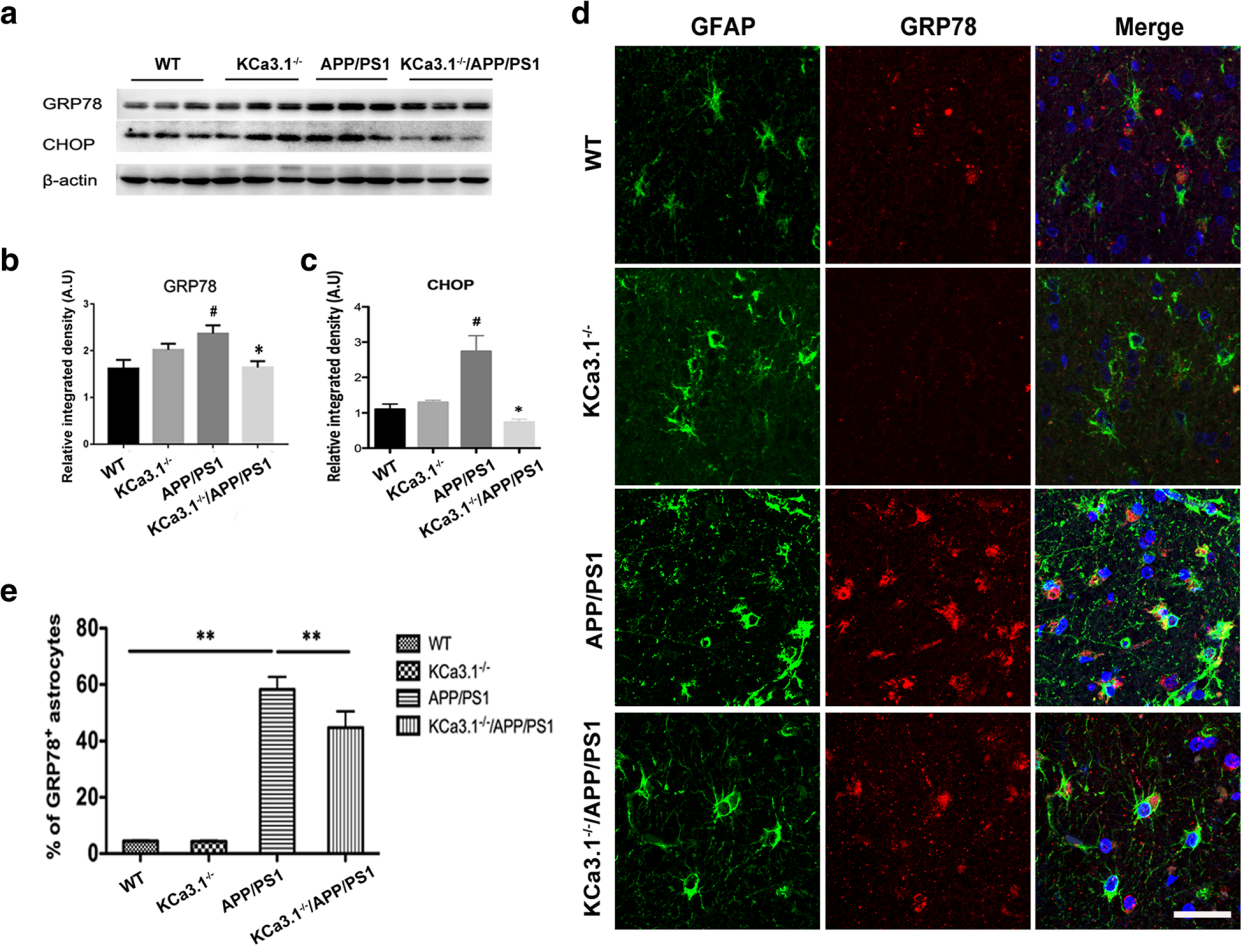
KCa3.1 contributes to increased ER stress in APP/PS1 mice. **a** Western blot analysis of GRP78 and CHOP protein levels in hippocampal extracts of 15-month-old WT, KCa3.1^−/−^, APP/PS1, and KCa3.1^−/−^/APP/PS1 mice. **b**, **c** Data are presented as the mean ± SEM (*n* = 3–5). The OD values of GRP78 (**b**) and CHOP (**c**) were normalized to that of β-actin. #*p* < 0.05 vs. WT, *p < 0.05 vs. APP/PS1 (one-way ANOVA followed by the Dunnett’s multiple comparison test). **d** Double immunofluorescence staining of GFAP with GRP78 in CA1 areas of the mouse hippocampus. DAPI (blue) was used to label nuclei. **e** Quantification of the percentage of GRP78^+^ cells colabeled with GFAP. **p < 0.01 vs. control group (one-way ANOVA followed by the Dunnett’s multiple comparison test) (*n* = 4). Scale bar: 25 μm. *WT* wild-type

Western blot analysis was conducted to identify changes in the expressions patterns of GRP78 and CHOP in the brains of 15-month-old APP/PS1 and KCa3.1^−/−^/APP/PS1 mice, as compared with WT and KCa3.1^−/−^ controls. Similar to previous results in AD patients (Fig. 1a, b), GRP78 and CHOP levels were significantly increased in APP/PS1 mice, as compared to WT and KCa3.1^−/−^ mice (*p* < 0.05, Fig. 4a–c), while the expression levels of GRP78 and CHOP were significantly decreased in KCa3.1^−/−^/APP/PS1 mice, as compared to APP/PS1 mice (*p* < 0.05, Fig. 4a–c).

It was reported that upregulation of GRP78 and phosphorylation of eIF2α in response to Aβ-induced ER stress contributed to RA in the AD mouse model and primary cultured astrocytes [25]. We then investigated whether KCa3.1 gene deletion could prevent changes in GRP78 expression levels in the astrocytes of APP/PS1 mice. To compare the response in astrocytes specifically, immunofluorescence was used to quantify GRP78 levels in GFAP^+^ astrocytes in the hippocampi of WT, KCa3.1^−/−^, APP/PS1, and KCa3.1^−/−^/APP/PS1 mice. In the hippocampi of APP/PS1 mice, GRP78 expression was increased in GFAP^+^ astrocytes, as compared to that of WT controls (*p* < 0.01, Fig. 4d, e). Meanwhile, hippocampal GRP78 immunoreactivity levels of KCa3.1^−/−^/APP/PS1 mice were significantly decreased, as compared to that of APP/PS1 mice (*p* < 0.01, Fig. 4d, e).

### Gene deletion of KCa3.1 activated the AKT/mTOR pathway

The foregoing data suggest that KCa3.1 gene deletion prevented activation of the UPR and attenuated astrocytic ER stress in an in vivo AD model. However, the signaling intermediates linking KCa3.1 and astrocytic ER stress in AD remain unknown. Given the reported relationship between the phosphatidylinositol 3-phosphate kinase/Akt pathway and ER stress [29], we tested whether the AKT pathway is involved in KCa3.1-mediated astrocytes ER stress.

**Fig. 5:**
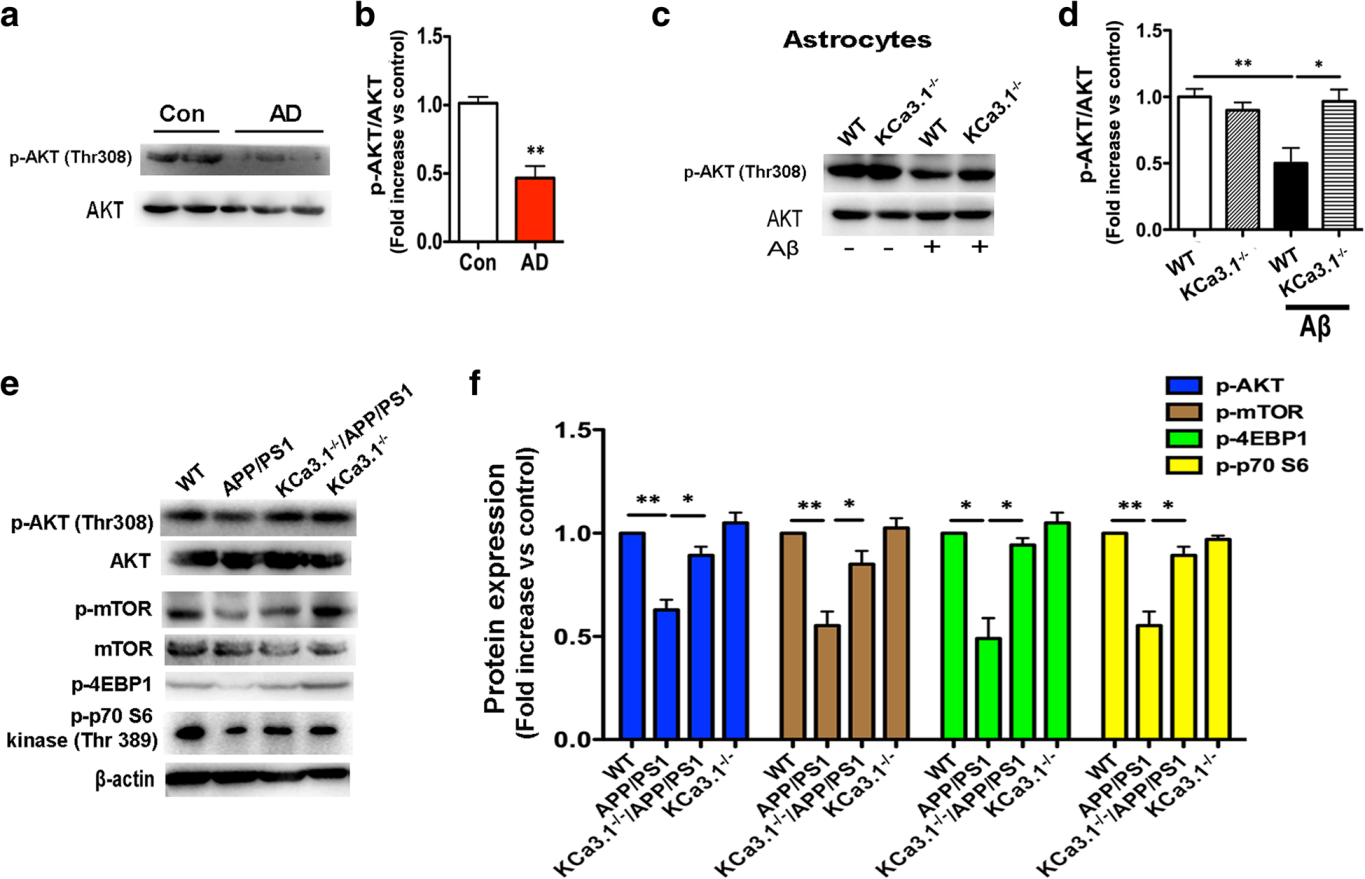
AKT modulation is crucial for KCa3.1-mediated ER stress in astrocytes. **a** Representative blots of p-AKT and total AKT from the hippocampi of postmortem human AD patients and age-matched controls. **b** Data are presented as the mean ± SEM (*n* = 3–5). The OD value of p-AKT was normalized to that of AKT. **p < 0.01 vs. control brains (unpaired, two-tailed Student’s *t* test). **c** Representative images of p-AKT and total AKT in KCa3.1^−/−^ astrocytes, responses to 5 μM Aβ 1 h vs. WT cells. **d** Mean values of p-AKT relative to AKT. Data are presented as the mean ± SEM (*n* = 3). *p < 0.05, **p < 0.01 vs. controls (one-way ANOVA followed by the Dunnett’s multiple comparison test). **e** Brain tissues from 15-month-old WT, KCa3.1^−/−^, APP/PS1, and KCa3.1^−/−^/APP/PS1 mice were subjected to SDS-PAGE, and immunoblotted with antibodies against p-AKT, total AKT, p-mTOR, total mTOR, p-4EBP1, and p-p70 S6. **f** Data are presented as the mean ± SEM (*n* = 4). The OD values of p-AKT and p-mTOR were normalized to those of AKT and mTOR, respectively. The OD values of p-4EBP1 and p-p70 S6 were normalized to that of β-actin. *p < 0.05, **p < 0.01 (one-way ANOVA followed by the Dunnett’s multiple comparison test). *Con* control, *WT* wild-type

Decreased phosphorylation of AKT (p-AKT) (Thr308) was observed in the brain tissues of AD patients (*p* < 0.01, Fig. 5a, b). In primary cultured astrocytes, Aβ treatment for 1 h significantly decreased p-AKT (Thr308) levels without inducing changes in total AKT (*p* < 0.01, Fig. 5c, d), while KCa3.1 gene deletion (KCa3.1^−/−^) inhibited the decrease in p-AKT (Thr308) after Aβ treatment (*p* < 0.05, Fig. 5c, d).

We then examined whether KCa3.1 gene deletion would activate the AKT/mTOR pathway in an in vivo AD model. Consistent with the in vitro results (Fig. 5c), p-AKT (Thr308) levels were decreased in the brain tissue of APP/PS1 mice (*p* < 0.01, Fig. 5e, f). KCa3.1 gene deletion in APP/PS1 mice (KCa3.1^−/−^/APP/PS1) attenuated p-AKT (Thr308) suppression (*p* < 0.05, Fig. 5e, f). Importantly, the phosphorylation of mTOR, a kinase that regulates cell survival, was decreased in the brain tissue of APP/PS1 mice (*p* < 0.01, Fig. 5e, f). mTOR suppression, in turn, suppressed its downstream proteins, including phosphorylated 4EBP1 (p-4EBP1) and p70 S6 kinase (p-p70 S6 kinase) (Thr389), while KCa3.1 gene deletion in APP/PS1 mice (KCa3.1^−/−^/APP/PS1) attenuated the suppression of mTOR phosphorylation (*p* < 0.05, Fig. 5e, f).

### Attenuation of neuronal loss in APP/PS1 mice lacking KCa3.1

**Fig. 6:**
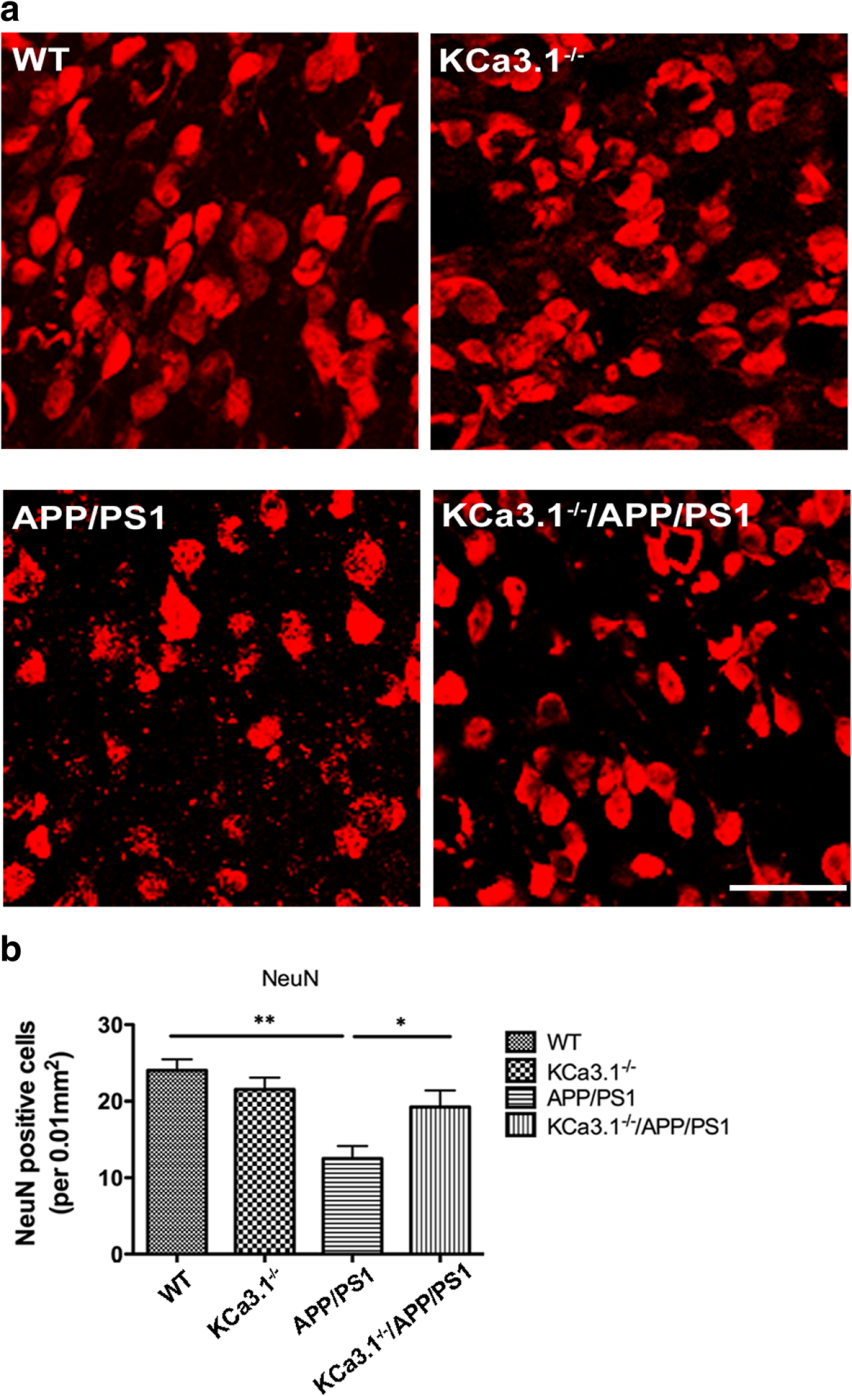
Neuronal loss is rescued in brains of KCa3.1^−/−^/APP/PS1 mice. **a** Immunofluorescence analysis of NeuN levels in the hippocampi of 15-month-old WT, KCa3.1^−/−^, APP/PS1, and KCa3.1^−/−^/APP/PS1 mice. **b** Quantification of neuron number/0.01 mm^2^ in the hippocampus (*n* = 6). Data are presented as the mean ± SEM. *p < 0.05, **p < 0.01 (one-way ANOVA followed by Dunnett’s post-hoc test). Scale bars: 25 μm

It was reported that blockade of KCa3.1 significantly reduced neuronal loss and memory deficits in both APP/PS1 mice and SAMP8 mice [11, 15]. Given that neuronal loss and decreased expression of synaptic proteins are likely correlated with the severity of AD [30], we compared expression levels of the neuron marker NeuN in the brain tissues of APP/PS1 mice with those of KCa3.1^−/−^/APP/PS1 mutants. Immunostaining analysis revealed that NeuN-positive neuron levels were decreased in the CA1 areas of the hippocampi of APP/PS1 mice, as compared with that in the WT group, while KCa3.1 gene deletion in APP/PS1 mice (KCa3.1^−/−^/APP/PS1) attenuated the decrease of NeuN staining (*p* < 0.05, Fig. 6a, b).

### Decreased glial activation and neuroinflammation in APP/PS1 mice lacking KCa3.1

**Fig. 7:**
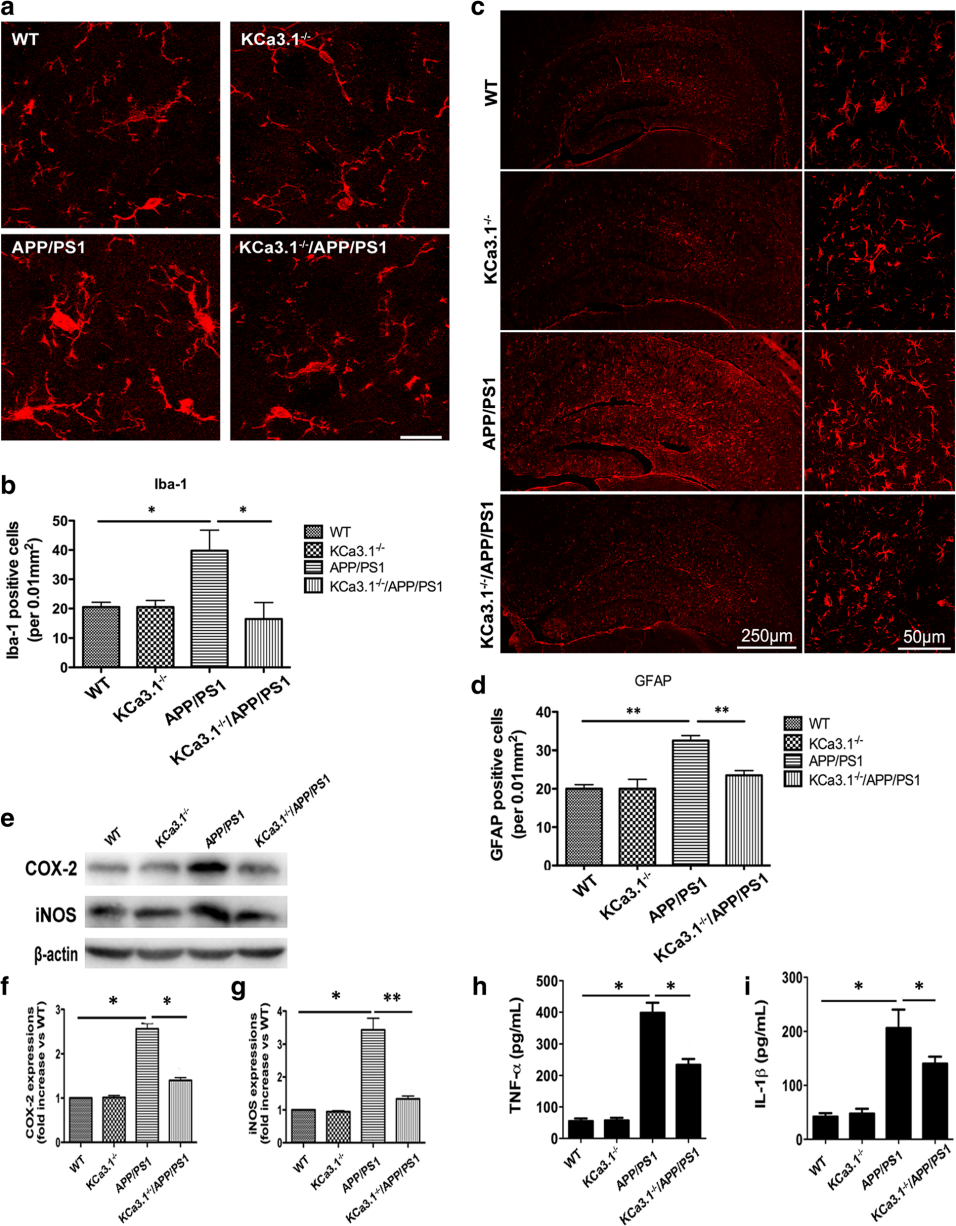
Decreased neuroinflammation in brains of KCa3.1^−/−^/APP/PS1 mice. **a** Levels of activated microglia in CA1 areas of the mouse hippocampus were analyzed by immunostaining of the microglia marker Iba1. **b** Quantification of activated microglia number/0.01 mm^2^ in the hippocampus (*n* = 3). Data are presented as the mean ± SEM. *p < 0.05 (one-way ANOVA followed by Dunnett’s post-hoc test). Scale bars: 25 μm. **c** Levels of reactive astrocytes in the CA1 area of the mouse hippocampus were analyzed by immunostaining of the astrocyte marker GFAP. **d** Quantification of reactive astrocytes number/0.01 mm^2^ in the hippocampus (*n* = 3). Data are presented as the mean ± SEM. *p < 0.05 (one-way ANOVA followed by Dunnett’s post-hoc test). Scale bars: 25 μm. **e**–**i** Gene deletion of KCa3.1 attenuated expression and release of inflammatory mediators in the brains of KCa3.1^−/−^/APP/PS1 mice. **e**–**g** Western blots showing protein expressions of COX-2 (**e**, **f**) and iNOS (**e**, **g**) proteins. **f**, **g** Data are presented as the mean ± SEM (*n* = 3). *p < 0.05, **p < 0.01 (One-way ANOVA followed by the Dunnett’s multiple comparison test). **h**, **i** Measurement of TNF-α (**h**) and IL-1β (**i**) released by ELISA in homogenated cortex of WT, KCa3.1^−/−^, APP/PS1, and KCa3.1^−/−^/APP/PS1 mice. Data represent mean ± SEM. *p < 0.05 (one-way ANOVA followed by the Dunnett’s multiple comparison test)

Both reactive astrocytes and microglial activation are predominant pathological features of AD [31]. Immunostaining of the RA marker GFAP was measured to determine whether KCa3.1 gene deletion affects RA in APP/PS1 mice. Immunostaining of GFAP^+^ astrocytes was increased in the brains of APP/PS1 mice, as compared with WT mice (*p* < 0.05, Fig. 7a, b), while KCa3.1 gene deletion in APP/PS1 mice (KCa3.1^−/−^/APP/PS1) attenuated the increase in immunostaining of GFAP^+^ astrocytes (*p* < 0.05, Fig. 7a, b).

We also investigated whether KCa3.1 gene deletion attenuates microglial activation in APP/PS1 mice by measuring expression levels of the microglia marker Iba1. Our data show that the content of Iba1^+^ microglia was increased in the brains of APP/PS1 mice, as compared with that of WT mice (*p* < 0.01, Fig. 7c, d). KCa3.1 gene deletion in APP/PS1 mice (KCa3.1^−/−^/APP/PS1) attenuated the increase of Iba1^+^ microglia immunostaining (*p* < 0.01, Fig. 7c, d). Both the RA and microglia activation results suggest that, while brains of APP/PS1 mice show increased glial responses, these responses are attenuated in the brains of KCa3.1^−/−^/APP/PS1 mice.

KCa3.1 deficiency in KCa3.1^−/−^/APP/PS1 mice attenuated the upregulation of COX-2 (Fig. 7e, f) and iNOS (Fig. 7e, g), compared with APP/PS1 mice. Levels of TNF-α and IL-1β in brain homogenates from WT, KCa3.1^−/−^, APP/PS1, and KCa3.1^−/−^/APP/PS1 group mice were measured by ELISA experiments (Fig. 7h, i). Levels of both TNF-α and IL-1β released were attenuated in the KCa3.1^−/−^/APP/PS1 group compared with the APP/PS1 group. Together, these findings indicate that elimination of KCa3.1 ameliorated the pathological hallmarks of AD in KCa3.1^−/−^/APP/PS1 mice, such as neuronal loss, microglial activation, RA, and neuroinflammation.

### KCa3.1 elimination in APP/PS1 mice rescues spatial memory deficits

**Fig. 8:**
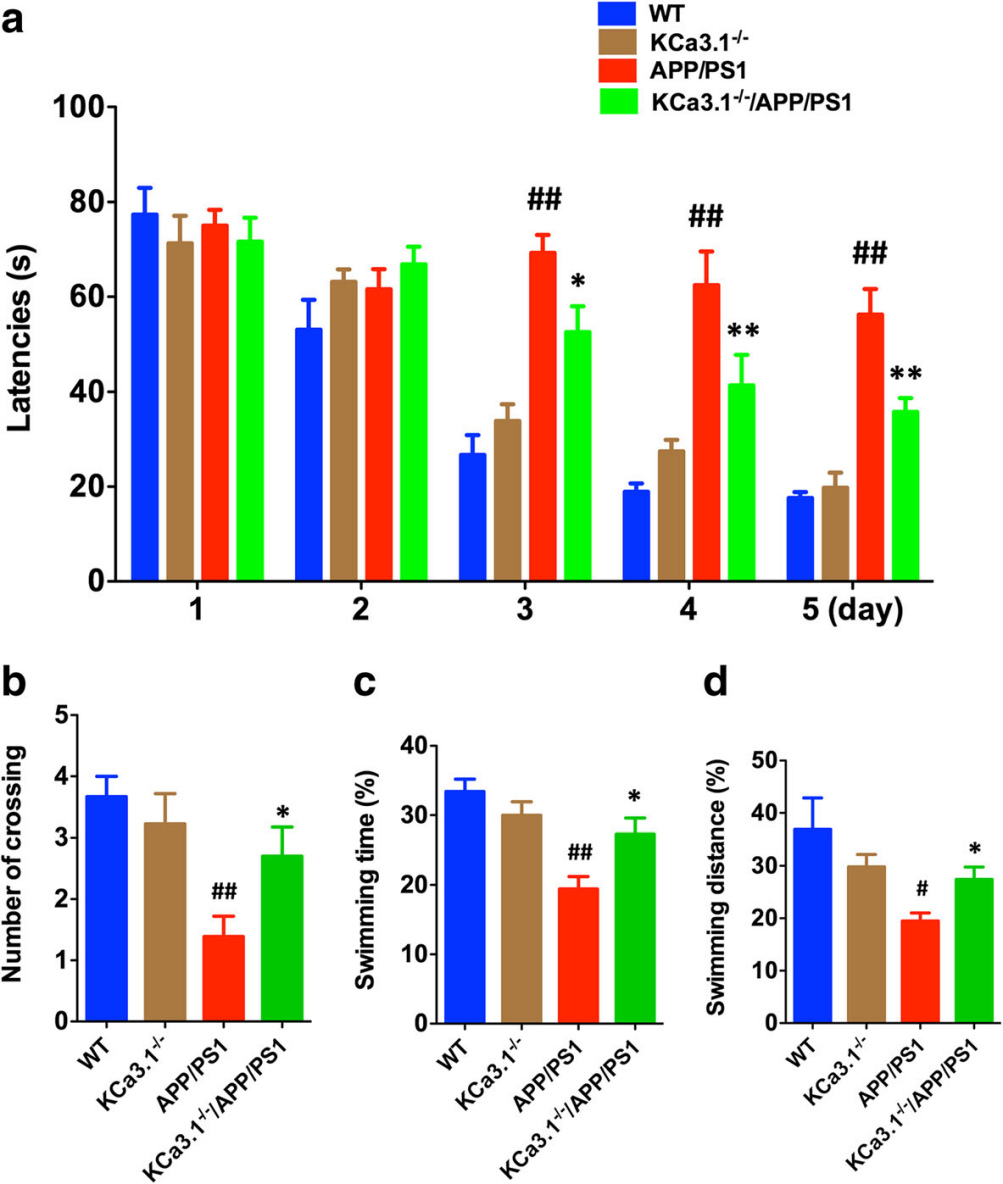
Elimination of KCa3.1 in APP/PS1 mice rescues spatial memory deficits in the MWM test. MWM testing of 15-month-old WT, KCa3.1^−/−^, APP/PS1, and KCa3.1^−/−^/APP/PS1 mice was performed as described in the “Materials and methods” section. **a** Escape latency. **b** Number of crossing the target quadrant by each group during the probe trials (no platform). **c** Percentage of swimming time spent in the target quadrant by each group during the probe trials (no platform). **d** Percentage of swimming distance spent in the target quadrant by each group during the probe trials (no platform). Data are presented as the mean ± SEM (*n* = 10–12). #*p* < 0.05, ##*p* < 0.01 vs. WT mice. *p < 0.05, **p < 0.01 vs. APP/PS1 mice. *WT* wild-type

In this study, the MWM spatial memory test was used to determine whether loss of KCa3.1 had any effect on cognitive deficits in APP/PS1 mice. With the use of a hidden platform during the MWM test, 15-month-old APP/PS1 mice showed memory deficits, as compared with WT mice (*p* < 0.01, Fig. 8a). Furthermore, the KCa3.1^−/−^/APP/PS1 mice showed improved spatial learning and memory, as compared with the APP/PS1 mice (Fig. 8a). In the spatial probe trial without an escape platform, significantly more KCa3.1^−/−^/APP/PS1 mice were able to cross the target quadrant, as compared to APP/PS1 mice (*p* < 0.05, Fig. 8b), and spent more time and swam for greater distances in the target quadrant (*p* < 0.05, Fig. 8c, d). In conclusion, the MWM test results indicate that APP/PS1 mice present significant spatial memory deficits and that elimination of KCa3.1 channels ameliorates these deficits.

## Discussion

The importance of KCa3.1 channels to CNS function is underscored by a series of studies implicating KCa3.1 in various diseases, including stroke/ischemia [32, 33], AD [11, 15], traumatic brain injury [34], and spinal cord injury [35]. These studies showed that upregulation of KCa3.1 activity can profoundly influence CNS pathology. Here, we report that KCa3.1 expression was markedly associated with ER stress and UPR in both Aβ-stimulated primary astrocytes and brain lysates of AD patients and APP/PS1 AD mice. Gene deletion or blockade of KCa3.1 attenuated SOCE-induced Ca^2+^ overload and ER stress, which regulates the AKT/mTOR pathway in RA to protect neurons from RA-induced neurotoxicity. Elimination of KCa3.1 can attenuate ER stress, neuronal loss, gliosis, neuroinflammation, and memory deficits observed in KCa3.1^−/−^/APP/PS1 mice. These data suggest that KCa3.1 presents an effective therapeutic target in AD.

Astrocytes perform brain homeostatic maintenance within the CNS and communicate with neighboring neurons and glial cells via [Ca^2+^]_i_ signals triggering the gliotransmitters release [3, 36–40]. SOCE channels, composed of STIM1 and Orai1 (pore-forming proteins), can be activated by the depletion of Ca^2+^ stores in the ER [7]. SOCE, which is regulated by STIM1/2 and Orai1, plays an essential role in the activation of non-excitable cells, including astrocytes and microglia, via triggering Ca^2+^ influx [8]. STIM1/2 and Orai1 were identified as key molecules of SOCE in cortical and spinal astrocytes. SOCE plays an important role in the production of the cytokines tumor necrosis factor-α and interluekin-6 in spinal astrocytes in response to lipopolysaccharides [27].

Dysfunctional astrocytic Ca^2+^ signaling is involved in many pathological states, such as epilepsy and ischemia [41]. Vincent et al. reported that astrocytic Ca^2+^ homeostasis is disrupted in AD. Disruption of Ca^2+^ signaling of astrocytes was found both in the transgenic mouse models of AD in vivo and in Aβ-stimulated primary astrocytes in vitro [5, 6, 42, 43]. ER stress plays an important role in the pathophysiology of various neurodegenerative diseases, including AD [44, 45]. Much evidence indicates a relationship between ER stress and Aβ-induced cytotoxicity in the post-mortem brain tissue of AD patients and AD mouse models [45, 46]. Aβ severely disrupts ER function and induces ER stress in neurons and glial cells [25, 47]. Stimulation with Aβ oligomers deregulated ER Ca^2+^ homeostasis in neurons and astrocytes, which resulted in misfolding of proteins in the ER. Excessive and prolonged activation of ER stress may initiate apoptosis by regulation of several key proteins, including CHOP, caspase-12, and JNK, which can further aggravate Aβ cytotoxicity [48]. ER stress is reportedly linked with some inflammation signaling networks, suggesting that ER stress activation may be involved in the neuroinflammatory response [49]. In the present study, we demonstrated that Aβ induced ER stress in astrocytes in vitro and observed upregulation of CHOP and the ER-resident chaperone GRP78 [50, 51]. Gene deletion and pharmacological blockade of KCa3.1 were shown to attenuate the upregulation of UPR hallmarks.

APP/PS1 mice present the prodromal phase of AD, reflecting memory and spatial learning deficits, as assessed by performance in the MWM test [52]. Soluble and plaque-associated Aβ peptides, which are present starting at 6 months of age in APP/PS1 transgenic mice, induce ER stress and sequent phosphorylation of PERK and eIF2a, which are closely associated with the pathogenesis of AD [53]. The results of the present study also suggest that elimination of KCa3.1 ameliorated the pathological hallmarks of AD in KCa3.1^−/−^/APP/PS1 mice, such as neuronal loss, microglial activation, and RA. Although the mechanisms underlying the attenuation of memory deficits by elimination of KCa3.1 are not well known, they are likely associated with the decreased pathological markers noted in KCa3.1^−/−^/APP/PS1 mice, as compared with APP/PS1 mice. Similar to AD patients, phosphorylation of GRP78 and eIF2α is upregulated in the brains of APP/PS1 mice [54]. APP/PS1 mice also showed decreased expression of NeuN and increased expression of Iba1 and GFAP, suggesting the presence of neuronal loss, microglial activation, and RA. Brain slices of KCa3.1^−/−^/APP/PS1 mice showed significantly lower levels of these markers than those of APP/PS1 mice, indicating that KCa3.1 gene deletion might involve in ER stress and, thereby, attenuate neuronal loss and glia inflammation in AD mice.

Our data also demonstrated that KCa3.1 regulates ER stress via the downstream AKT/mTOR signaling pathway during RA in AD. We observed that Aβ decreased the activation of AKT by decreasing AKT phosphorylation in primary astrocytes. Interestingly, KCa3.1 elimination prevented Aβ-mediated loss of AKT activation via upregulation of AKT phosphorylation at Thr308. Growing evidence indicates that the mTOR signaling pathway plays a vital role during apoptosis and autophagy. Our results show that Aβ represses the phosphorylation of AKT, mTOR, p70 S6 kinase, and 4EBP1, and that a decrease in the phosphorylation of AKT led to UPR activation. The postmortem brain samples from patients with and without AD were also evaluated. The results indicate that KCa3.1 expression and activation of UPR-associated proteins are increased in the brains of AD patients.

## Conclusions

Overall, these data indicate that KCa3.1 plays an important role in the process of Ca^2+^ homeostasis in the ER and that KCa3.1 upregulation involves in the activation of the UPR pathway, which subsequently leads to neurodegeneration in AD.

## Abbreviations

ATF6: Activating transcription factor 6
CHOP: CCAAT enhancer-binding protein homologous protein
DAPI: 4′,6-Diamidino-2-phenylindole
EGTA: Ethylene glycol-bis(β-aminoethyl ether)-*N*,*N*,*N*′,*N*′-tetraacetic acid
GFAP: Glial fibrillary acidic protein
GRP78: 78 kDa glucose-regulated protein
IRE1: Inositol-requiring enzyme 1
KCa3.1: Intermediate-conductance calcium-activated potassium channel
PERK: PKR-like ER kinase
ROS: Reactive oxygen species
SAMP8: Senescence-accelerated mouse prone 8
SDS-PAGE: Sodium dodecyl sulfate-polyacrylamide gel electrophoresis
SOCE: Store-operated Ca^2+^ entry
TRAM-34: 1-((2-Chlorophenyl) (diphenyl) methyl)-1H–pyrazole
UPR: Unfolded protein response
